# Alopecia Universal após Tratamento com Sinvastatina e Ezetimiba: Impactos na Família

**DOI:** 10.36660/abc.20220187

**Published:** 2022-10-05

**Authors:** Ferhat Ozyurtlu, Nurullah Cetin

**Affiliations:** 1 Special Grand Medical Hospital Department of Cardiology Manisa Turquia Special Grand Medical Hospital – Department of Cardiology , Manisa – Turquia; 2 Celal Bayar University Faculty of Medicine Department of Cardiology Manisa Turquia Celal Bayar University Faculty of Medicine , Department of Cardiology , Manisa – Turquia

**Keywords:** Alopecia, Doença Autoimune, Hipercolesterolemia, Atorvastatina/efeitos adversos, Combinação de Ezetimiba e Sinvastatina/efeitos adversos, Genética

## Abstract

A alopecia areata (AA) é uma doença autoimune que se desenvolve no couro cabeludo ou em outras partes do corpo. A alopecia universal, que é uma forma rara de alopecia areata, é caracterizada pela perda de pelos que afeta todo o corpo. Nos dois pacientes apresentados, o tratamento com atorvastatina foi iniciado com o diagnóstico de hipercolesterolemia, mas, quando as metas de valores não foram alcançadas, foi iniciado o tratamento com uma combinação de sinvastatina e ezetimiba. Depois de um período de tratamento com sinvastatina e ezetimiba, o distúrbio de AA, o qual começou com a perda de cabelo no couro cabeludo, espalhou pelo corpo todo e se transformou em alopecia universal. Embora as estatinas possam causar alopecia com reações autoimunes, elas geralmente são utilizadas no tratamento da alopecia, por seus efeitos imunomoduladores.

## Caso 1

**Figura 1:**
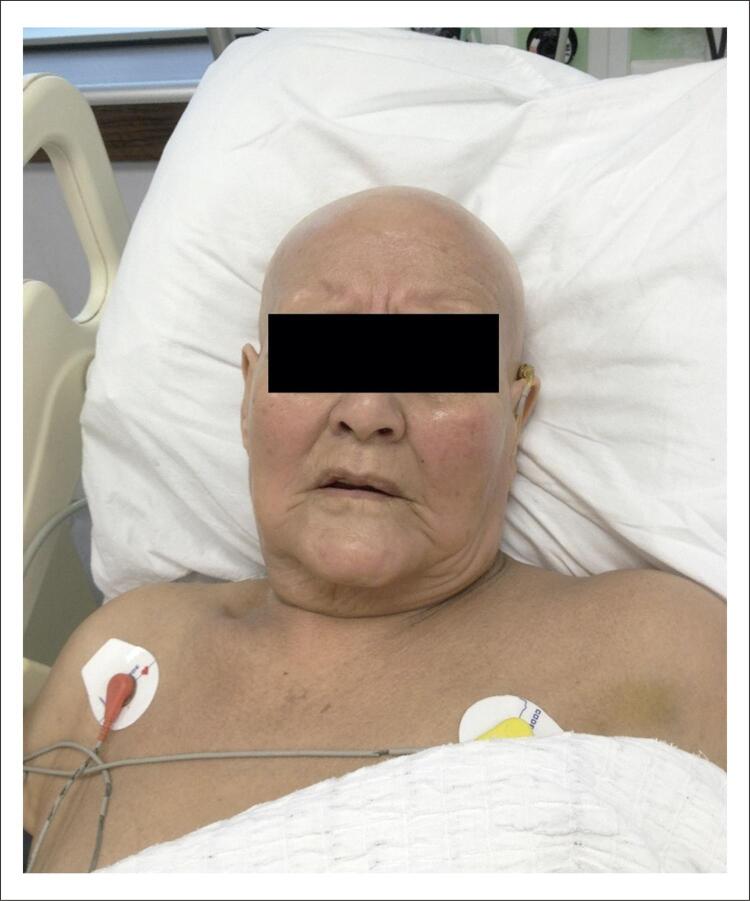
Paciente com alopecia universal, caso 1.

Uma paciente de 69 anos foi acompanhada em nossa clínica com o diagnóstico de insuficiência cardíaca e doença arterial coronariana. A paciente tinha histórico de hipercolesterolemia. Seu escore Ductch (escore clínico por hipercolesterolemia familiar, com um diagnóstico definitivo > 8 pontos) foi calculado como 12 pontos. Os parâmetros lipídeos obtidos em nossa clínica foram: colesterol total de 380 mg/dl, lipoproteína de baixa densidade (LDL) de 299 mg/dl, lipoproteína de alta densidade (HDL) de 62 mg/dl, e triglicérides 93 mg/dl. No histórico médico da paciente, descobriu-se que a paciente havia usado comprimidos de atorvastatina de 40 mg (Lipitor, Pfizer) por 6 meses, há 5 anos, mas o tratamento foi alterado por uma combinação de sinvastatina de 40 mg e ezetimiba de 10 mg (Inegy 10/40, Merck, Sharp & Dohme) porque os valores-alvo não podiam ser alcançados. Isso resultou na queda, primeiramente de cabelo, seguida da queda de sobrancelhas, cílios, e dos pelos axilares e pubianos em 2 meses. A alopecia universal aumentou em aproximadamente 6 meses ( Figura 1 ).

## Caso 2

**Figura 2:**
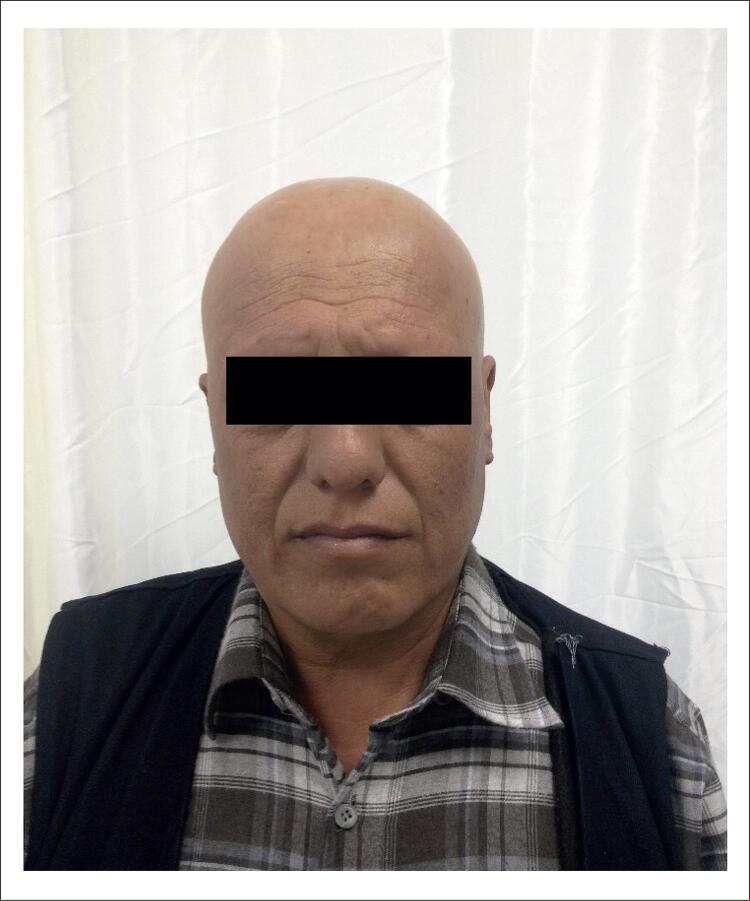
Paciente com alopecia universal, caso 2.

O segundo caso foi do filho de 45 anos de idade da paciente, que também apresentou alopecia universal ( Figura 2 ). Seu escore de Dutch foi calculado em 14 pontos. Os parâmetros lipídeos do paciente foram os seguintes: colesterol total de 382 mg/dl, colesterol LDL de 305 mg/dl, colesterol HDL de 57 mg/dl, e triglicérides 102 mg/dl. Esse paciente foi tratado com comprimidos de atorvastatina de 40 mg (Lipitor, Pfizer) simultaneamente com sua mãe. Quando se identificou que o tratamento não era eficaz após 5 meses, o tratamento usando a combinação de 40 mg de sinvastatina e 10 mg de ezetimiba (Inegy, Merck/Sharp & Dohme) foi iniciado. Da mesma forma, após o início do tratamento, observou-se a queda, primeiramente do cabelo, seguida da queda de sobrancelhas, cílios, e dos pelos axilares e pubianos em 2 meses, e a alopecia universal aumentou em aproximadamente 6 meses ( Figura 2 ). Considerando-se que pode ser uma patologia relacionada a medicamentos, a aplicação de remédios foi interrompida. Entretanto, não se observou remissão. Ambos os casos recusaram o tratamento dermatológico para o tratamento da alopecia.

## Discussão

AA é uma forma de alopecia não cicatricial com queda de cabelos em placas no couro cabeludo e queda de pelos em outros locais. Ela pode ocorrer em qualquer idade, e é observada com mais frequência na segunda e na quarta décadas de vida. Geralmente, ela é observada em ambos os sexos com a mesma frequência. A incidência dessa situação que pode ser aceita como relativamente comum é 0,15%. ^1^ Embora a patogênese da doença ainda não seja totalmente conhecida, ela é uma doença autoimune e, além dos fatores genéticos, os fatores ambientais, tais como infecção e stress psicológico, também desempenham um papel importante na evolução da doença. A incidência familiar de 10%–20% corrobora a ativação genética da doença, o qual aumenta para 50% em gêmeos monozigóticos. ^2^ Entretanto, a alopecia universal é uma forma rara de AA, que é definida como a queda de cabelos e de pelos corporais. Ela constitui 7%–30% de todos os casos de AA. ^3^

As estatinas são os principais agentes terapêuticos para o tratamento da hipercolesterolemia. Efeitos além daqueles pretendidos durante a evolução de um agente são chamados de efeitos pleiotrópicos. A redução de isoprenóides circulantes e a inativação de proteínas sinalizadoras resulta em efeitos pleiotrópicos de estatinas, tais como, efeitos anti-inflamatórios, antioxidantes, antiproliferativos e imunomoduladores, estabilidade de placa e inibição da agregação de plaquetas. ^4^ As estatinas executam seus efeitos imunomoduladores que são pleiotrópicos, via moléculas de MHC-II, e células T auxiliar 1 e T auxiliar 2. ^5^ Também é sabido que as células T auxiliar 1 e T auxiliar 2 têm funções específicas na AA. ^6^ Uma reação autoimune que desencadeia um mecanismo de estatinas pode causar a liberação de autoantígenos por apoptose e, portanto, uma resposta de autoanticorpos. Entretanto, ela desencadeia a ativação dos linfócitos T causando uma mudança no teor de colesterol da estrutura lipídica da membrana. O resultado é a reação da célula T auxiliar 2 que leva à produção de autoanticorpos por células B. ^7 , 8^ Na literatura, há casos em que a sinvastatina foi usada no tratamento da alopecia devido a seus efeitos imunomoduladores. ^9^ Além disso, sabe-se que as estatinas causam lesão hepática, como miopatia autoimune e hepatite autoimune. ^10 , 11^ Embora a queda de cabelo tenha sido relatada entre os efeitos colaterais incomuns das estatinas nas bulas, há um caso em que queda de cabelo relacionada a atorvastatina foi relatada na literatura. ^12^ Ezetimiba diminui a absorção do colesterol obtido pela dieta. Nenhum caso de alopecia causado pela monoterapia com ezetimiba foi relatado. Além disso, foi relatado um caso de hepatite autoimune relacionada à combinação de ezetimiba e sinvastatina. Entretanto, só é possível especular se o fator que causou isso estava relacionado à estatina ou ao ezetimiba. ^13^ Embora estatina, ezetimiba e sua combinação sejam usados no tratamento da alopecia devido a seus efeitos imunomoduladores, ironicamente, em nossos casos, acredita-se que a combinação foi temporariamente associada ao início da alopecia em ambos os casos, e pode ter contribuído para a doença. Além disso, observa-se que fatores genéticos têm um grande papel nos casos em que o mesmo medicamento causa a alopecia e se torna o tratamento para ela.
