# Supplementary material for: Alopecia Universal após Tratamento com Sinvastatina e Ezetimiba: Impactos na Família
Source: Arq Bras Cardiol. 2022 Oct 5;119(4):631–3. [Article in Portuguese] doi: 10.36660/abc.20220187 (PMC9563873; doi:10.36660/abc.20220187)
Supplement: Translation

# Translation: Alopecia Universalis after Treatment with Simvastatin and Ezetimibe: Affects on Family

**Authors:** Ferhat Ozyurtlu, Nurullah Cetin

**Affiliations:** 1 Turkey Special Grand Medical Hospital – Department of Cardiology, Manisa- Turkey; 2 Turkey Celal Bayar University Faculty of Medicine, Department of Cardiology, Manisa – Turkey

**Keywords:** Alopecia, Autoimmune Diseases, Hypercholesterolemia, Atorvastatin/adverse effects, Ezetimibe, Simvastatin Drug Combination/adverse effects, Genetic

## Abstract

Alopecia areata (AA) is an autoimmune disease that grows in the scalp or in other parts of the body. Alopecia universalis, which is a rare form of alopecia areata, is characterized by a loss of hair that affects the entire body. In the two patients presented in this study, atorvastatin treatment was implemented, with the diagnosis of hypercholesterolemia; however, when the target values were not reached, a combination of simvastatin and ezetimibe was implemented. After a period of simvastatin/ezetimibe treatment, the AA disorder, which began with hair loss on the scalp, spread to the entire body and turned into Alopecia Universalis. Although statins can cause alopecia with autoimmune reactions, they are generally used in the treatment of alopecia due to their immunomodulatory effects.

## Case 1

A 69-year-old female patient was followed up in our clinic with the diagnosis of heart failure and coronary artery disease. The patient had a history of hypercholesterolemia. Her Dutch score (clinical score for familial hypercholesterolemia, with a definite diagnosis > 8 points) was calculated as 12 points. The lipid parameters obtained in our clinic were: total cholesterol of 380 mg/dl, low-density lipoprotein (LDL) cholesterol of 299 mg/dl, high-density lipoprotein (HDL) cholesterol of 62 mg/dl, and triglycerides of 93 mg/dl. In the patient’s medical history, it was learned that the patient had used 40 mg atorvastatin tablets (Lipitor, Pfizer) for 6 months, 5 years prior, but the treatment was changed to a combination of 40 mg simvastatin and 10 mg ezetimibe (Inegy 10/40, Merck, Sharp & Dohme), since the target values could not be reached. This resulted in the patient losing of hair first from the head and then from the eyebrow, eyelashes, and the hair on the axillar and pubic parts of the body in 2 months. Alopecia universalis increased in approximately 6 months ( Figure 1 ).

**Figure 1:** Alopecia universalis patient, case 1.

## Case 2

The second case was the 45-year-old son of the female patient, who also showed alopecia universalis ( Figure 2 ). His Dutch score was calculated as 14 points. The patient’s lipid parameters were as follows: total cholesterol of 382 mg/dl, LDL cholesterol of 305 mg/dl, HDL cholesterol of 57 mg/dl, and triglycerides of 102 mg/dl. This patient was treated with 40 mg atorvastatin tablets (Lipitor, Pfizer) simultaneously with his mother. When the treatment proved to be ineffective after 5 months, the combination treatment of 40 mg simvastatin and 10 mg ezetimibe (Inegy, Merck/Sharp & Dohme) was implemented. Likewise, after the treatment began, first the hair on the head, then eyebrow, eyelashes, and the hair on the axillar and pubic part of the body began to fall out in 2 months, and alopecia universalis increased in approximately 6 months ( Figure 2 ). Considering that it can be a medicine-related pathology, the application of medicine was stopped; however, no remission was observed in the table. Both cases refused to receive dermatological treatment for the treatment of alopecia.

**Figure 2:** Alopecia universalis patient, case 2.

## Discussion

AA is a non-scarring form of alopecia with a patchy loss of hair in the scalp and elsewhere. It can occur at any age, and it is more frequently observed in the second and fourth decades. Generally, it is observed in both sexes with the same frequency. The incidence of this situation that can be accepted as relatively common is 0.15%.^1^ Although the pathogenesis of the disease is not completely known, it is an autoimmune disease and apart from genetic factors; the environmental factors such as infection and psychological stress also play an important role in the development of this disease. The family incidence of 10%–20% supports the genetic activation of the disease, which increases to 50% for monozygotic twins.^2^ However, alopecia universalis is a rare form of AA, which is defined as the loss of all hair of the head and the body. It constitutes 7%–30% of all AA cases.^3^

The statins are the main therapeutic agents for treating hypercholesterolemia. Effects other than the intended effect during the development of an agent are called pleiotropic effects. Reduction in circulating isoprenoids and the inactivation of signaling proteins result in pleiotropic effects of statins, such as anti-inflammatory, antioxidant, antiproliferative, and immunomodulatory effects, plaque stability, and the inhibition of platelet aggregation.^4^ Statins execute their immunomodulatory effects, which are pleiotropic, via MHC-II moles, T helper 1 cells, and T helper 2 cells.^5^ It is also well-known that in AA, T helper 1 and T helper 2 cells play specific roles.^6^ An autoimmune reaction triggering mechanism of statins can cause apoptosis to release autoantigens and hence an autoantibody response. However, this triggers T-lymphocyte activation by causing a change in the cholesterol content of the membrane lipid structure. The result is a T helper 2 reaction that results in autoantibody production by B cells.^7 , 8^ In the literature, there are cases in which simvastatin was used in alopecia treatment because of its immunomodulator effects.^9^ Along with this, it is also well-known that statins cause liver damage, such as autoimmune myopathy and autoimmune hepatitis.^10 , 11^ Although hair loss has been reported among the uncommon side effects of statins in the prospectus information, there is one case in which atorvastatin-related hair loss was reported in the literature.^12^ Ezetimibe decreases the absorption of cholesterol obtained from the diet. No alopecia case caused by ezetimibe monotherapy has been reported. Moreover, an Ezetimibe/Simvastatine combination related to an autoimmune hepatitis case was reported. However, only a speculation can be done as to whether or not the factor causing this was statin-related or ezetimibe-related.^13^ Although statin, ezetimibe, and their combination were used in alopecia treatment because of their immunomodulator effects, ironically, in our cases, it is believed that the combination was temporally associated with the beginning of alopecia in both cases, and may have contributed to the condition. Furthermore, it was observed that genetic factors play a broad role in which the same medicine both causes alopecia and becomes the treatment for alopecia.
